# AGREEing on Nutritional Management of Patients with CKD—A Quality Appraisal of the Available Guidelines

**DOI:** 10.3390/nu13020624

**Published:** 2021-02-15

**Authors:** Dimitra Rafailia Bakaloudi, Lydia Chrysoula, Kalliopi Anna Poulia, Evangelia Dounousi, Vassilios Liakopoulos, Michail Chourdakis

**Affiliations:** 1 Laboratory of Hygiene, Social & Preventive Medicine and Medical Statistics, School of Medicine, Faculty of Health Sciences, Aristotle University of Thessaloniki, 54124 Thessaloniki, Greece; dimitrabakaloudi@gmail.com (D.R.B.); lydian285@gmail.com (L.C.); mhourd@gapps.auth.gr (M.C.); 2 Department of Nutrition and Dietetics, Laiko General Hospital, 11527 Athens, Greece; lpoulia@gmail.com; 3 Department of Nephrology, Faculty of Medicine, School of Health Sciences, University of Ioannina, 45110 Ioannina, Greece; evangeldou@gmail.com; 4 Division of Nephrology and Hypertension, 1st Department of Internal Medicine, AHEPA Hospital, School of Medicine, Faculty of Health Sciences, Aristotle University of Thessaloniki, 54636 Thessaloniki, Greece

**Keywords:** AGREE, chronic kidney disease, clinical practice, guidelines, nutrition support, nutritional status, recommendations

## Abstract

Chronic kidney disease (CKD) is an important public health issue with increasing prevalence worldwide. Several clinical practice guidelines have been recently published regarding the nutritional management of CKD patients. The purpose of the present study is to evaluate the quality of the published guidelines and provide recommendation for future updates. PubMed, Scopus and Google Scholar were searched for relevant guidelines and 11 clinical practice guidelines were finally included. Guidelines developed by the American Society for Parenteral and Enteral nutrition (ASPEN), the Dietitians Association of Australia (DAA), the German Society for Nutritional Medicine (DGEM), the European Best Practice Guidelines (EBPG), the European Dialysis and Transplantation Nurses Association-European Renal Care Association (EDTNA-ERCA), the European Society for Clinical Nutrition and Metabolism (ESPEN), the Andalusian Group for Nutrition Reflection and Investigation (GARIN) group, the National Kidney foundation-Kidney Disease Outcomes Quality Initiative (KDOQI), the Italian Society of Nephrology-Association of Dieticians-Italian Association of Hemodialysis, Dialysis and Transplant (SIN-ANDID-ANED), and the Renal Association were assessed using the Appraisal of Guidelines for Research and Evaluation (AGREE) II tool. Guidelines by KDOQI, ESPEN, and DAA were of moderate quality and the rest of them were low-quality guidelines. Our study demonstrates gaps related to the development of guidelines and therefore greater emphasis on methodological approaches is recommended. AGREE II tool can be useful to improve quality of guidelines.

## 1. Introduction

Chronic kidney disease (CKD) remains a strong cause of adverse health outcomes, with a constant rise in prevalence and a recorded 41.5% global increase from 1990 to 2017 in all age-mortality rates [1]. It was estimated that in 2017, 697.5 million people were diagnosed with any stage of CKD, which is equivalent to a 9.1% prevalence worldwide [1]. Furthermore, it is expected that the number of patients receiving renal replacement therapy (RRT) will be doubled by 2030, with the highest increase predicted for Asia and North America. This will eventually lead to a higher total cost of treatment, especially for patients with comorbidities, resulting in an economic burden on healthcare systems [2,3]. In order to avoid such a trend and avert an additional financial pressure on health systems, the development of low-cost strategies towards the prevention and reduction in the adverse outcomes of CKD is essential, if not critical [4].

It has been well established that lifestyle and dietary modifications are a cost-effective strategy, producing both cost- and health-related benefits [5,6,7]. In addition, improved nutritional status and compliance with the medical nutrition guidance has been positively associated with a better quality of life (QoL) and cognitive function, lower risk of complications (e.g., hypertension, dyslipidemia, obesity, cardiovascular, the progression of CKD, etc.), reduced risk of malnutrition and overall CKD-related symptoms [8,9,10]. Therefore, it is essential for health professionals to comprehend the importance and complexity of the provision of nutritional support in these patients and emphasize the formulation and delivery of appropriate nutritional treatment followed by regular monitoring [11,12]. Such actions require the systematic development of clinical guidelines in order to ensure patients’ safety and provision of high-quality nutritional care [13].

Clinical practice guidelines can be defined as evidence-based statements and recommendations aiming to enhance health outcomes of patients and simultaneously assist health professionals in their decision-making processes and implementation of individualized patient care [14]. So far, a number of clinical guidelines have been published by several scientific societies, as well as international institutions and organizations, aiming to ensure the best nutritional management for patients with CKD. However, clinical practice guidelines often present high heterogeneity and a number of questions over their validity, reliability, applicability in clinical practice, transparency, and methodological rigor have been raised. In addition, there has not been any attempt to assess the quality and the development process of nutritional practice guidelines for patients with CKD. The aim of this study is to critically appraise the quality of existing clinical practice guidelines on the nutritional management of CKD and to identify their limitations and discrepancies with regard to structure, methodology, and development. Moreover, the provision of suggestions for potential areas of quality improvement is another scope of the present work, using the revised Appraisal of Guidelines for Research and Evaluation tool (AGREE II), an international tool that has been widely used in the past 10 years for the quality assessment of medical guidelines, as the appraisal instrument [15].

## 2. Materials and Methods

Systematic searches on PubMed, Scopus and Google scholar databases were performed (until 12th of November 2020) using the follow search string (guidelines OR recommendations OR statements) AND (CKD OR renal failure OR chronic kidney disease OR kidney failure) AND (nutrition OR diet OR nutritional management) for the PubMed database; this was modified accordingly for the other databases (see Supplementary File 1). From a total 5687 results and after removing duplicates and irrelevant studies, 297 studies were screened for eligibility. Inclusion criteria were recently published nutritional guidelines for patients with CKD, developed by scientific societies and/or associations. Irrelevant recommendations, hospital internal guidelines or recommendations referring only to subjects <18 years old were excluded. Finally, 11 clinical practice guidelines published from 2003 until 2020 were retrieved [16,17,18,19,20,21,22,23,24,25,26]. Clinical practice guidelines by the American Society for Parenteral and Enteral nutrition (ASPEN) [16], the Dietitians Association of Australia (DAA) [17], the German Society for Nutritional Medicine (DGEM) [18], the European Best Practice Guidelines (EBPG) [19], the European Dialysis and Transplantation Nurses Association-European Renal Care Association (EDTNA-ERCA) [20], the European Society for Clinical Nutrition and Metabolism (ESPEN) [21,22], the Andalusian Group for Nutrition Reflection and Investigation (GARIN) group [23], the National Kidney foundation-Kidney Disease Outcomes Quality Initiative (KDOQI) [24], the Italian Society of Nephrology-Association of Dieticians-Italian Association of Hemodialysis, Dialysis and Transplant (SIN-ANDID-ANED) [25], and the Renal Association [26] were reviewed and evaluated using the AGREE II tool. [15] Translations of original clinical practice guidelines were excluded. The latest updated version of each guideline was selected for guidelines with previous published versions. Characteristics of eligible clinical practice guidelines that were included can be found in Table 1.

### Guideline Appraisal

Four authors, including medical doctors, nutritionists, and dietitians (DB, LC, KAP and MC), independently evaluated and appraised the 11 practice guidelines with the use of the AGREE II tool [15]. Each reviewer ratings can be found in Supplementary File 2. The AGREE tool was first developed in 2003 in an effort to provide researchers with an instrument that would aid in the quality assessment, reporting, evaluation, and improvement of clinical practice guidelines within various health settings [27]. The AGREE II tool, which is the updated version, consists of a reporting checklist including 23 items categorized under the following six main quality domains: 1. scope and purpose, 2. stakeholder involvement, 3. rigor of development, 4. clarity of presentation, 5. Applicability, and 6. editorial independence, as well as two more items under “overall assessment” concerning the overall quality of the guideline and recommendations on whether or not it should be used in practice [15]. Each item is rated using a 7-point scale (1 = strongly disagree, 7 = strongly agree). Finally, the total score for each domain is calculated by summing up all ratings for all items of each domain and then expressed as a percentage of the maximum possible score in each domain, minus the minimum possible score as follows:

(1) $$\frac{Obtained\;score-Minimun\;possible\;score}{Maximum\;possible\;score-Minimum\;possible\;score}\;\times \;100$$

In order to ensure the consistency regarding the evaluation of the included guidelines, specific quality thresholds were set. High-quality and “recommended” guidelines were those with scores ≥70% for all domains; moderate quality and “recommended with modifications” guidelines were those with scores ≥70% in three to five domains; low-quality and not recommended guidelines were those with scores ≥70% in none of the domains or up to two domains.

## 3. Results

Results of the evaluations of the 11 clinical practice guidelines can be found in Table 2. Three clinical practice guidelines were found to be of moderate quality [17,21,24]. High-quality clinical guidelines for the nutritional management of patients with CKD were not found according to the parameters of the AGREE II tool. The lowest score, i.e., one point per reviewer, is equal to 14.3% in terms of percentage in the AGREE tool II and reflects either the poor description of the relevant information or its total absence from the guideline.

### 3.1. Scope and Purpose Domain

The majority of the clinical practice guidelines received high ratings in the domain of “scope and purpose”, while six of them received a score ≥70% [17,18,21,22,23,24]. Four guidelines received low scores [16,20,25,26] and the EBPG guideline received the lowest [19].

### 3.2. Stakeholder Involvement Domain

In this domain, none of the included guidelines were highly rated by the four reviewers. Five studies had rates of 40–70%, which translates into a moderate quality [16,17,18,24,25], whereas in the remaining six studies had rate scores <40%, leading to low-quality characterization [19,20,21,22,23,26]. Item 2b, “target population’s views and preferences”, was sufficiently stated in only two guidelines (DAA and SIN-ANDIN-ANED) [17,25]. Moreover, item 2a, which refers to the expertise and the roles of members taking part in the guideline development group, was only reported in the KDOQI guidelines [24].

### 3.3. Rigor of Development

Τhe results for the domain “rigor of development” varied. This domain evaluates all stages of the methodological development of each guideline review as well as information on the update process. The highest score can be observed in the KDOQI guidelines [24], whereas in the other guidelines many items of this domain were not included [16,17,19,20,21,22,25,26] or were not satisfactorily described [18,23].

### 3.4. Clarity of Presentation

Regarding the clarity of presentation, the results were satisfactory. Clinical practice guidelines developed by DGEM received the highest scores (100) [18]. High scores were also reported in seven clinical practice guidelines which scored >70% [17,19,20,21,22,23,24] and three guidelines were determined to be of moderate quality [16,25,26]. None of the guidelines received a low score for this domain.

### 3.5. Applicability

As per the domain of “Applicability”, none of the included clinical practice guidelines received a total score ≥70%. Moreover ASPEN, SIN-ANDID-ANED and the Renal Association received scores of zero as they failed to mention this aspect in their manuscripts [16,25,26].

### 3.6. Editorial Independence

In this domain, the reviewers appraised the included clinical practice guidelines according to the information provided for the funding and the authors’ conflicts of interest during the guideline development. In eight guidelines, exclusive funding statements were found [16,17,18,20,21,23,24,25], but information on whether this funding influenced the content of guidelines was not always reported (i.e., DGEM, EDTNA-ERCA, and SIN-ANDID-ANED guidelines [18,20,25]). In the ASPEN and DAA guidelines, no funding existed [16,17]. Moreover, statements of competing interest were not found in the ASPEN and GARIN guidelines [16,23].

**Figure 1:**
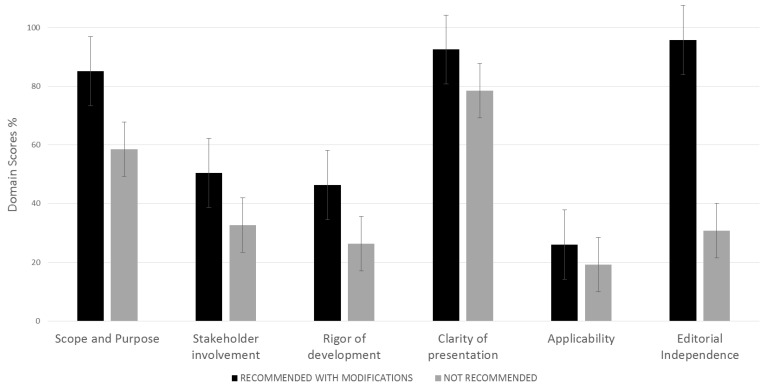
Average quality score of each domain between recommended with modifications and not recommended guidelines.

The average quality score of each domain between recommended with modifications and not recommended guidelines can be seen in Figure 1 and a summary of the AGREE II tool results of each domain can be found in Supplementary Figure S1.

## 4. Discussion

The aim of the present study was to assess the quality of development of 11 nutritional guidelines for patients with CKD using the AGREE II tool [15]. According to our results, scores between the domains of each guideline differed significantly. Clinical guidelines developed by the KDOQI received the highest rating (≥70% in four domains) [24], followed by ESPEN (EN) and DAA guidelines (≥70% in three domains) [17,21] and were recommended with modifications by the reviewers. Moreover, clinical guidelines by ASPEN, DGEM, EBPG, EDTNA–ERCA, ESPEN (PN), GARIN, SIN–ANDID–ANED, and the Renal Association [16,18,19,20,22,23,25,26] were characterized by a low overall quality and thus were not recommended based on the AGREE II tool [15].

Based on the AGREE II reported items, there are several fields in the quality of guidelines that could be improved. In general, sufficient evidence for the purpose and scope (Domain 1) can be found in the guidelines studied. Regarding this domain, EPBG [19] and the Renal Association [26] failed to clearly describe the population covered by the guidelines. Clarifying purpose and scope can prohibit the use of guidelines for a nonappropriate group of patients.

As for the stakeholder involvement (Domain 2), more information about expertise, geographical location as well as the role of each member in guideline development could improve the significance of EDTNA-ERCA, GARIN, and the Renal Association guidelines [20,23,26], as an emphasis on multidisciplinary approaches could have a positive impact on the quality of the guidelines. Population experiences and expectations were not adequately described in the included guidelines and this should be considered for the future updated versions. Moreover, target users’ information is missing in the EBPG [19] guideline, which should be clearly defined so as the clinician can recognize whether the guideline is appropriate for his/her use.

As per rigor of development (Domain 3), the intense heterogeneity that was found between the scores (Table 2) proves the existence of significant gaps regarding the guidelines’ methodological development. Only in the KDOQI [24] guideline description and methods of evidence selection were described. This is the domain in which several modifications are needed (i.e., better description of research methodology, report of included/excluded criteria, strengths and potential limitations of guideline development, link between scientific evidence and recommendations, etc.) in order to improve the quality of the existing clinical practice guidelines.

In most of the guidelines examined, recommendations were clearly presented (Domain 4), facilitating the easy accessibility and application by the clinician, something that is important especially in acute settings. Tables, summarizing boxes, bold font, and/or numbers were used in all analyzed nutritional guidelines and/or key recommendations. Moreover, only in the SIN-ANDID-ANED guideline [25], the item of different options for management in patients with CKD was not satisfactorily described, a parameter that indicates that a different approach according to stage and the severity of CKD is needed, as claimed by the majority of included guidelines. An important parameter that should be highlighted is that although the AGREE II tool includes the item regarding different options for management, consideration of the patient’s condition in the very beginning of their treatment is missing. None of the 11 guidelines examined included satisfactory statements for the first approach according to the condition of the patient. For example, novel, ketogenic diets, prescribed by trained physicians, could prove helpful for obese patients with mild CKD [28,29], but in recently published guidelines, special recommendations for this group of patients were not stated.

As per applicability, an important domain of the AGREE II instrument (Domain 5), all nutritional guidelines failed to adequately report facilitators and barriers and/or provide tools, advice, and potential resources that can affect implementation in clinical practice; consequently, in all guidelines, the total obtained score for this domain was <70%. The best described item of this domain was that of monitoring, which was only not applicable for ASPEN, SIN-ANDID-ANED, and the Renal Association clinical practice guidelines [16,25,26].

Finally, the evidence of funding and/or conflicts of interest were not informative in all cases. Only in the DAA [17] clinical practice guideline was editorial independence (Domain 6) entirely described. The fact that funding body information and/or competing interest statements are not clearly and adequately described can be associated with several biases regarding the content of recommendations. As the AGREE II tool can sufficiently identify the problematic areas in the clinical practice guidelines, it is highly recommended that this tool should be used by guidelines developers as a checklist with a view to keeping structure and adequacy in guideline development methodology and to ensuring the high quality of the guidelines produced.

Our study has several strengths. First, to the best of our knowledge, this is the first study that compares and evaluates the quality of the clinical practice guidelines in nutritional management of patients with CKD. Moreover, the assessment and rating of the eligible guidelines was performed independently by a multidisciplinary group of four reviewers, including medical doctors and dieticians. Furthermore, it is the first time that the AGREE II tool has been used for the quality evaluation of the nutritional practice guidelines for CKD.

One of the limitations of our study is that we included guidelines in English, Spanish, and German and we may have excluded other published guidelines in different languages. Moreover, the fact that nine out of 11 guidelines included in our study came from European regions could mean that the results of our study might be difficult to be applied to patients in other regions. In addition, the appraisal tool does not provide standard cut offs for the evaluation of scores and this could affect the interpretation of our results. Furthermore, it should be clarified that in this study, only the critical appraisal of the quality development of the guidelines was performed without any assessment of the quality of the guidelines’ content.

## 5. Conclusions

The quality of the development of the majority of the nutritional clinical practice guidelines on management of CKD patients it is not satisfactory. The guidelines that were the best in terms of quality according to the AGREE II tool were the KDOQI, DAA, and ESPEN (EN), ensuring a better quality of information, even if they all require modifications in future update processes [17,21,24]. Additionally, as several gaps in methodology development were identified in most of the guidelines, the following updates should incorporate these changes in order to ameliorate their quality. Editorial independence should be described in detail in order to eliminate bias. AGREE II can be a useful tool in the evaluation of quality of clinical practice guidelines.

## Figures and Tables

**Table 1:** Characteristics of included guidelines.

No.	Developer ^#^	Year	Region	Intended Population	Scope/Grading System	Number of References	Disciplines Involved in the Group
1	ASPEN	2010	USA	Adult patients with AKI and CKD	Provision of nutrition support to patients with AKI and CKD/Level of evidence I–V	64	Physicians, nurses, pharmacologists, dieticians
2	DAA *	2006	Australia	Adults with CKD	Dietetic management of adult patients with CKD/Level of evidence I–IV	19	Dietitians, nephrologists, renal nurses
3	DGEM *	2015	Germany	Adults with AKD, CKD, dialysis and transplant patients	Nutrition support and metabolic management in the care of patients with renal dysfunction/No info	77	Physicians
4	EBPG	2007	Europe	Adult patients on dialysis	Prevalence, diagnosis and treatment of malnutrition of patients on dialysis/No info	337	Dietitians, nephrologists
5	EDTNA-ERCA	2003	Europe	Adults with CKD	Support healthcare professionals on the task of nutrition advice of renal patients/No info	53	Physicians, dietitians, nurses
6	ESPEN	2006	Europe	Adult patients with renal failure and patients on dialysis	Recommendations for the use of ONS and TF in nephrology patients/Grade A–C	72	No info
7	ESPEN	2009	Europe	Adult patients with renal failure and patients on dialysis	Indications for parenteral nutrition in renal patients with malnutrition/Grade A–C	123	Physicians, dietitians
8	GARIN	2018	Spain	Adult patients with CKD stages 1–5 (including patients on dialysis)	define dietary characteristics for adults with renal diseases/GRADE-ASPEN	96	Endocrinology and nutrition specialists
9	KDOQI *	2020	International	Adult patients with CKD stages 1–5, (including patients on dialysis), and patients with functional kidney transplant	Provision of MNT guidelines for patients with CKD to assess, prevent, and treat protein-energy wasting, mineral and electrolyte disorders, and other metabolic comorbid conditions associated with CKD/Grade A-D	531	Physicians, registered dietitians- nutritionists, researchers, methodologists with expertise in the renal and nutrition field
10	Renal Association	2019	UK	Adult patients with CKD stages 4 and 5 (including dialysis)	Prevalence, diagnosis and treatment of undernutrition in chronic kidney disease/The modified GRADE system	75	No information
11	SIN-ANDID-ANED	2018	Italy	Adult patients with advanced renal insufficiency	Promotion of a successful and safe implementation of nutritional treatment for CKD patients/No info	167	Nephrologists, dietitians, patients

CKD: Chronic kidney disease, NA: Not Applicable, AKI: Acute Kidney Injury, AKD: Acute Kidney Disease, GRADE: Grading of Recommendations Assessment, Development and Evaluation, MNT: Medical Nutrition Therapy, ONS: Oral Nutritional Supplements, TF: Tube feeding, UK: United Kingdom. ASPEN: American Society for Parenteral and Enteral Nutrition, DAA: Dietitians Association of Australia, DGEM: Deutsche Gesellschaft für Ernährungsmedizin-German Society for Nutritional Medicine, EBPG: European Best Practice Guidelines, EDTNA-ERCA: European Dialysis and Transplantation Nurses Association/European Renal Care Association, ESPEN: European Society for Clinical Nutrition and Metabolism, KDOQI: Kidney Disease Outcomes Quality Initiative, SIN-ANDID-ANED: Italian Society of Nephrology-Association of Dieticians-Italian Association of Hemodialysis, Dialysis and Transplant, and GARIN: Andalusian Group for Nutrition Reflection and Investigation. ^#^ All developers are medical/nutritional societies. * Updated version of guidelines.

**Table 2:** Appraisal of Guidelines for Research and Evaluation (AGREE) II results for clinical practice guidelines.

Domains	Clinical Practice Guidelines										
	ASPEN [16]	DAA [17]	DGEM [18]	EBPG [19]	EDTNA-ERCA [20]	ESPEN (EN) [21]	ESPEN (PN) [22]	GARIN [23]	KDOQI [24]	SIN-ANDID-ANED [25]	The Renal Association [26]
**1. SCOPE AND PURPOSE**	**34.7**	**88.9**	**88.9**	**0.00**	**62.5**	**77.8**	**81.9**	**86.1**	**88.9**	**55.6**	**5.6**
a. Overall objectives	75.0	85.7	82.1	14.3	75.0	71.4	89.3	89.3	**89.3**	60.7	25.0
b. Health questions	17.9	96.4	89.3	14.3	53.6	85.7	89.3	89.3	**100**	67.9	14.3
c. Population to apply	39.3	89.3	**100**	14.3	75.0	85.7	75.0	89.3	92.0	57.1	17.9
**2. STAKEHOLDER INVOLVEMENT**	**62.5**	**63.9**	**44.4**	**16.7**	**34.7**	**36.1**	**12.5**	**15.3**	**51.4**	**43.1**	**18.1**
a. Guideline development group	85.7	75.0	64.3	53.6	32.1	53.6	42.9	39.3	**100**	71.4	32.1
b. Views and preferences of target population	28.6	**71.4**	14.3	17.9	32.1	14.3	14.3	14.5	17.9	53.6	14.3
c. Users of guidelines	**89.3**	60.7	78.6	14.3	67.9	67.9	17.9	28.6	57.1	28.6	42.9
**3. RIGOR OF DEVELOPMENT**	**31.3**	**30.2**	**48.9**	**21.9**	**13.0**	**36.5**	**18.2**	**47.4**	**72.4**	**3.6**	**33.3**
a. Research methodology	32.1	32.1	82.1	14.3	14.3	17.9	14.3	50.0	**96.4**	14.3	60.7
b. Selecting criteria	14.3	17.9	14.2	14.3	14.3	14.3	17.9	75.0	**100**	14.3	14.3
c. Strengths and limitations of evidence	14.3	14.3	14.3	28.6	14.3	14.3	17.9	85.7	**85.7**	14.3	42.9
d. Formulating methods	46.4	42.9	50.0	14.3	17.9	39.3	25.0	32.1	**82.1**	14.3	53.6
e. Health benefits, side effects and risks stated in recommendations	75.0	*14.3*	**92.9**	82.1	67.9	75.0	75.0	57.1	32.1	25.0	25.0
f. Explicit link of recommendations	89.3	*82.1*	92.9	82.1	46.4	96.4	60.7	96.4	**100**	28.6	71.4
g. Review of guideline	42.9	*57.1*	14.3	14.3	14.3	28.6	14.3	28.6	**100**	14.3	60.7
h. Updating procedure	14.3	*60.7*	**89.3**	14.3	14.3	78.6	14.3	14.3	14.3	14.3	14.3
**4. CLARITY OF PRESENTATION**	**52.8**	**84.7**	**100**	**86.1**	**91.7**	**94.4**	**91.7**	**94.4**	**98.6**	**33.3**	**68.1**
a. Specific and unambiguous recommendations	67.9	83.3	**100**	89.3	96.4	89.3	**100**	96.4	**100**	46.4	85.7
b. Different options for management	14.3	*92.9*	**100**	75.0	85.7	96.4	96.4	89.3	96.4	25.0	60.7
c. Identifiable key recommendations	96.4	96.4	**100**	**100**	96.4	**100**	82.1	**100**	**100**	57.1	71.4
**5. APPLICABILITY**	**0.0**	**11.5**	**12.5**	**27.1**	**36.5**	**4.2**	**33.3**	**25.0**	**62.5**	**0.0**	**0.0**
a. facilitators and barriers of application	14.3	14.3	14.3	17.9	32.1	14.3	14.2	14.3	**28.6**	14.3	14.3
b. guideline implementation advice and tools	14.3	21.4	14.3	60.7	67.9	14.3	67.9	57.1	**96.4**	14.3	14.3
c. Resource implications	14.3	21.4	14.3	28.5	28.6	14.3	14.3	17.9	**57.1**	14.3	14.3
d. Monitoring and/or auditing criteria	14.3	39.3	57.1	42.9	53.6	28.6	75.0	53.6	**89.3**	14.3	14.3
**6. EDITORIAL INDEPENDENCE**	**35.4**	**100**	**47.9**	**0.00**	**36.5**	**95.8**	**6.3**	**39.6**	**91.7**	**50.0**	**47.9**
a. Views of funding body	75.0	**100**	21.4	14.3	17.9	96.4	14.3	82.1	85.7	42.9	14.3
b. Conflicts of interests	14.3	**100**	89.3	14.3	17.9	96.4	25.0	14.3	**100**	71.4	96.4
**OVERALL QUALITY**	Low	Moderate	Low	Low	Low	Moderate	Low	Low	Moderate	Low	Low
**RECOMMENDATIONS**											
Recommended											
With modifications		**X**				**X**			**X**		
Not recommended	**X**		**X**	**X**	**X**		**X**	**X**		**X**	**X**

All results are presented as percentage scores. Total score of each domain and the highest score of each category are presented in bold font. EN: Enteral nutrition; PN: Parenteral nutrition. AoND: Academy of Nutrition and Dietetics, ASPEN: American Society for Parenteral and Enteral Nutrition, DAA: Dietitians Association of Australia, DGEM: Deutsche Gesellschaft für Ernährungsmedizin-German Society for Nutritional Medicine, EBPG: European Best Practice Guidelines, EDTNA-ERCA: European Dialysis and Transplantation Nurses Association/European Renal Care Association, ESPEN: European Society for Clinical Nutrition and Metabolism, KDOQI: Kidney Disease Outcomes Quality Initiative, GARIN: Andalusian Group for Nutrition Reflection and Investigation.

## Data Availability

Not applicable.

## Supplementary Materials

The following are available online at https://www.mdpi.com/2072-6643/13/2/624/s1, Supplementary File 1: Search strategy, Supplementary File 2: Ratings of each reviewer, Supplementary Figure S1: AGREE II tool results of each domain.

## Abbreviations

ASPEN	American Society for Parenteral and Enteral Nutrition
CKD	Chronic kidney disease
DAA	Dietitians Association of Australia
EBPG	European best practice guidelines
EDTNA-ERCA	European Dialysis and Transplantation Nurses Association-European Renal Care Association
ESPEN	European Society for Clinical Nutrition and Metabolism
KDOQI	Kidney disease outcomes quality initiative
MNT	Medical nutrition therapy
QoL	Quality of life
RRT	Renal replacement therapy
SIN-ANDID-ANED	Italian Society of Nephrology-Association of Dieticians-Italian Association of Hemodialysis, Dialysis and Transplant
