# Supplementary material for: AGREEing on Nutritional Management of Patients with CKD—A Quality Appraisal of the Available Guidelines
Source: Nutrients. 2021 Feb 15;13(2):624. doi: 10.3390/nu13020624 (PMC7918946; doi:10.3390/nu13020624)
Supplement: Supplementary file 1 [file nutrients-13-00624-s001.zip › nutrients-1050150-supplementary materials/Supplementary File 1_Search strategy.docx]

**Supplementary File 1**: Search terms of electronic databases

**Pubmed (12/11/2020)**

(<https://www.ncbi.nlm.nih.gov/pubmed/>)

(guidelines OR recommendations OR statements) AND (CKD OR renal failure OR chronic kidney disease OR kidney failure) AND (nutrition OR diet OR nutritional management) – All fields

Results: 2364

**Scopus (12/11/2020)**

(<https://www.scopus.com/home.uri>)

TITLE-ABS-KEY ( ( guidelines OR recommendations OR statements ) AND ( ckd OR "renal failure" OR "chronic kidney disease" OR "kidney failure" ) AND ( nutrition OR diet OR "nutritional management" ) )

Results: 3312

**Google Scholar (12/11/2020)**

<(https://scholar.google.com/)>

allintitle: nutrition guidelines CKD

Results: 11

**Total number of studies: N= 5687**
