# Supplementary material for: AGREEing on Nutritional Management of Patients with CKD—A Quality Appraisal of the Available Guidelines
Source: Nutrients. 2021 Feb 15;13(2):624. doi: 10.3390/nu13020624 (PMC7918946; doi:10.3390/nu13020624)
Supplement: Supplementary file 1 [file nutrients-13-00624-s001.zip › nutrients-1050150-supplementary materials/Supplementary File 2.docx]

| **AGREE II DOMAINS** | **Guideline from advising bodies** | | | | |
| --- | --- | --- | --- | --- | --- |
|  | **ASPEN** | | | | |
| **Country** | **USA** | | | | |
| **Appraiser** | **Appraiser 1** | **Appraiser 2** | **Appraiser 3** | **Appraiser 4** | **TOTAL** |
| **1. SCOPE AND PURPOSE** |  |  |  |  |  |
| 1a | 6 | 5 | 5 | 5 | 21 |
| 1b | 1 | 1 | 2 | 1 | 5 |
| 1c | 2 | 2 | 4 | 3 | 11 |
| **TOTAL** | 9 | 8 | 11 | 9 | 37 |
| **TOTAL DOMAIN SCORE (%)** | **34,72** | | | | |
| **2. STAKEHOLDER INVOLVEMENT** | | | | | |
| 2a | 6 | 6 | 6 | 6 | 24 |
| 2b | 1 | 1 | 4 | 2 | 8 |
| 2c | 7 | 6 | 6 | 6 | 25 |
| **TOTAL** | 14 | 13 | 16 | 14 | 57 |
| **TOTAL DOMAIN SCORE (%)** | **62,5** | | | | |
| **3. RIGOUR OF DEVELOPMENT** | | | | | |
| 3a | 3 | 2 | 2 | 2 | 9 |
| 3b | 1 | 1 | 1 | 1 | 4 |
| 3c | 1 | 1 | 1 | 1 | 4 |
| 3d | 5 | 3 | 2 | 3 | 13 |
| 3e | 7 | 3 | 6 | 5 | 21 |
| 3f | 7 | 6 | 6 | 6 | 25 |
| 3g | 5 | 1 | 3 | 3 | 12 |
| 3h | 1 | 1 | 1 | 1 | 4 |
| **TOTAL** | 30 | 18 | 22 | 22 | 92 |
| **TOTAL DOMAIN SCORE (%)** | **31,25** | | | | |
| **4. CLARITY OF PRESENTATION** | | | | | |
| 4a | 5 | 3 | 6 | 5 | 19 |
| 4b | 1 | 1 | 1 | 1 | 4 |
| 4c | 6 | 7 | 7 | 7 | 27 |
| **TOTAL** | 12 | 11 | 14 | 13 | 50 |
| **TOTAL DOMAIN SCORE (%)** | **52,78** | | | | |
| **5. APPLICABILITY** | | | | | |
| 5a | 1 | 1 | 1 | 1 | 4 |
| 5b | 1 | 1 | 1 | 1 | 4 |
| 5c | 1 | 1 | 1 | 1 | 4 |
| 5d | 1 | 1 | 1 | 1 | 4 |
| **TOTAL** | 4 | 4 | 4 | 4 | 16 |
| **TOTAL DOMAIN SCORE (%)** | **0** | | | | |
| **6. EDITORIAL INDEPENDENCE** | | | | | |
| 6a | 4 | 5 | 7 | 5 | 21 |
| 6b | 1 | 1 | 1 | 1 | 4 |
| **TOTAL** | 5 | 6 | 8 | 6 | 25 |
| **TOTAL DOMAIN SCORE (%)** | **35,42** | | | | |

| **AGREE II DOMAINS** | **Guideline from advising bodies** | | | | |
| --- | --- | --- | --- | --- | --- |
|  | **DAA** | | | | |
| **Country** | **Australia** | | | | |
| **Appraiser** | **Appraiser 1** | **Appraiser 2** | **Appraiser 3** | **Appraiser 4** | **TOTAL** |
| **1. SCOPE AND PURPOSE** |  |  |  |  |  |
| 1a | 6 | 6 | 6 | 6 | 24 |
| 1b | 7 | 7 | 6 | 7 | 27 |
| 1c | 7 | 7 | 5 | 6 | 25 |
| **TOTAL** | 20 | 20 | 17 | 19 | 76 |
| **TOTAL DOMAIN SCORE (%)** | **88,89** | | | | |
| **2. STAKEHOLDER INVOLVEMENT** | | | | | |
| 2a | 6 | 4 | 6 | 5 | 21 |
| 2b | 5 | 6 | 4 | 5 | 20 |
| 2c | 4 | 6 | 3 | 4 | 17 |
| **TOTAL** | 15 | 16 | 13 | 14 | 58 |
| **TOTAL DOMAIN SCORE (%)** | **63,88888889** | | | | |
| **3. RIGOUR OF DEVELOPMENT** | | | | | |
| 3a | 3 | 3 | 1 | 2 | 9 |
| 3b | 1 | 1 | 2 | 1 | 5 |
| 3c | 1 | 1 | 1 | 1 | 4 |
| 3d | 2 | 3 | 4 | 3 | 12 |
| 3e | 1 | 1 | 1 | 1 | 4 |
| 3f | 6 | 5 | 6 | 6 | 23 |
| 3g | 3 | 4 | 5 | 4 | 16 |
| 3h | 5 | 3 | 5 | 4 | 17 |
| **TOTAL** | 22 | 21 | 25 | 22 | 90 |
| **TOTAL DOMAIN SCORE (%)** | **30,20833333** | | | | |
| **4. CLARITY OF PRESENTATION** | | | | | |
| 4a | 5 | 5 | 5 | 5 | 20 |
| 4b | 7 | 7 | 6 | 6 | 26 |
| 4c | 7 | 7 | 6 | 7 | 27 |
| **TOTAL** | 19 | 19 | 17 | 18 | 73 |
| **TOTAL DOMAIN SCORE (%)** | **84,72222222** | | | | |
| **5. APPLICABILITY** | | | | | |
| 5a | 1 | 1 | 1 | 1 | 4 |
| 5b | 1 | 2 | 2 | 1 | 6 |
| 5c | 2 | 1 | 2 | 1 | 6 |
| 5d | 2 | 3 | 3 | 3 | 11 |
| **TOTAL** | 6 | 7 | 8 | 6 | 27 |
| **TOTAL DOMAIN SCORE (%)** | **11,45833333** | | | | |
| **6. EDITORIAL INDEPENDENCE** | | | | | |
| 6a | 7 | 7 | 7 | 7 | 28 |
| 6b | 7 | 7 | 7 | 7 | 28 |
| **TOTAL** | 14 | 14 | 14 | 14 | 56 |
| **TOTAL DOMAIN SCORE (%)** | **100** | | | | |

| **AGREE II DOMAINS** | **Guideline from advising bodies** | | | | |
| --- | --- | --- | --- | --- | --- |
|  | **DGEM** | | | | |
| **Country** | **GERMANY** | | | | |
| **Appraiser** | **Appraiser 1** | **Appraiser 2** | **Appraiser 3** | **Appraiser 4** | **TOTAL** |
| **1. SCOPE AND PURPOSE** |  |  |  |  |  |
| 1a | 6 | 5 | 6 | 6 | 23 |
| 1b | 7 | 6 | 6 | 6 | 25 |
| 1c | 7 | 7 | 7 | 7 | 28 |
| **TOTAL** | 20 | 18 | 19 | 19 | 76 |
| **TOTAL DOMAIN SCORE (%)** | **88,89** | | | | |
| **2. STAKEHOLDER INVOLVEMENT** | | | | | |
| 2a | 6 | 4 | 4 | 4 | 18 |
| 2b | 1 | 1 | 1 | 1 | 4 |
| 2c | 5 | 6 | 5 | 6 | 22 |
| **TOTAL** | 12 | 11 | 10 | 11 | 44 |
| **TOTAL DOMAIN SCORE (%)** | **44,44444444** | | | | |
| **3. RIGOUR OF DEVELOPMENT** | | | | | |
| 3a | 6 | 6 | 5 | 6 | 23 |
| 3b | 1 | 1 | 1 | 1 | 4 |
| 3c | 1 | 1 | 1 | 1 | 4 |
| 3d | 3 | 4 | 3 | 4 | 14 |
| 3e | 7 | 7 | 5 | 7 | 26 |
| 3f | 6 | 6 | 7 | 7 | 26 |
| 3g | 1 | 1 | 1 | 1 | 4 |
| 3h | 7 | 6 | 6 | 6 | 25 |
| **TOTAL** | 32 | 32 | 29 | 33 | 126 |
| **TOTAL DOMAIN SCORE (%)** | **48,95833333** | | | | |
| **4. CLARITY OF PRESENTATION** | | | | | |
| 4a | 7 | 7 | 7 | 7 | 28 |
| 4b | 7 | 7 | 7 | 7 | 28 |
| 4c | 7 | 7 | 7 | 7 | 28 |
| **TOTAL** | 21 | 21 | 21 | 21 | 84 |
| **TOTAL DOMAIN SCORE (%)** | **100** | | | | |
| **5. APPLICABILITY** | | | | | |
| 5a | 1 | 1 | 1 | 1 | 4 |
| 5b | 1 | 1 | 1 | 1 | 4 |
| 5c | 1 | 1 | 1 | 1 | 4 |
| 5d | 4 | 3 | 4 | 5 | 16 |
| **TOTAL** | 5 | 6 | 7 | 8 | 28 |
| **TOTAL DOMAIN SCORE (%)** | **12,5** | | | | |
| **6. EDITORIAL INDEPENDENCE** | | | | | |
| 6a | 3 | 1 | 1 | 1 | 6 |
| 6b | 6 | 6 | 6 | 7 | 25 |
| **TOTAL** | 9 | 7 | 7 | 8 | 31 |
| **TOTAL DOMAIN SCORE (%)** | **47,91666667** | | | | |

| **AGREE II DOMAINS** | **Guideline from advising bodies** | | | | |
| --- | --- | --- | --- | --- | --- |
|  | **EBPG** | | | | |
| **Country** | **Europe** | | | | |
| **Appraiser** | **Appraiser 1** | **Appraiser 2** | **Appraiser 3** | **Appraiser 4** | **TOTAL** |
| **1. SCOPE AND PURPOSE** |  |  |  |  |  |
| 1a | 1 | 1 | 1 | 1 | 4 |
| 1b | 1 | 1 | 1 | 1 | 4 |
| 1c | 1 | 1 | 1 | 1 | 4 |
| **TOTAL** | 3 | 3 | 3 | 3 | 12 |
| **TOTAL DOMAIN SCORE (%)** | **0,00** | | | | |
| **2. STAKEHOLDER INVOLVEMENT** | | | | | |
| 2a | 5 | 5 | 2 | 3 | 15 |
| 2b | 2 | 1 | 1 | 1 | 5 |
| 2c | 1 | 1 | 1 | 1 | 4 |
| **TOTAL** | 8 | 7 | 4 | 5 | 24 |
| **TOTAL DOMAIN SCORE (%)** | **16,66666667** | | | | |
| **3. RIGOUR OF DEVELOPMENT** | | | | | |
| 3a | 1 | 1 | 1 | 1 | 4 |
| 3b | 1 | 1 | 1 | 1 | 4 |
| 3c | 2 | 1 | 3 | 2 | 8 |
| 3d | 1 | 1 | 1 | 1 | 4 |
| 3e | 7 | 5 | 5 | 6 | 23 |
| 3f | 5 | 6 | 6 | 6 | 23 |
| 3g | 1 | 1 | 1 | 1 | 4 |
| 3h | 1 | 1 | 1 | 1 | 4 |
| **TOTAL** | 19 | 17 | 19 | 19 | 74 |
| **TOTAL DOMAIN SCORE (%)** | **21,875** | | | | |
| **4. CLARITY OF PRESENTATION** | | | | | |
| 4a | 7 | 6 | 6 | 6 | 25 |
| 4b | 6 | 5 | 5 | 5 | 21 |
| 4c | 7 | 7 | 7 | 7 | 28 |
| **TOTAL** | 20 | 18 | 18 | 18 | 74 |
| **TOTAL DOMAIN SCORE (%)** | **86,11111111** | | | | |
| **5. APPLICABILITY** | | | | | |
| 5a | 1 | 1 | 2 | 1 | 5 |
| 5b | 4 | 4 | 5 | 4 | 17 |
| 5c | 2 | 1 | 3 | 2 | 8 |
| 5d | 3 | 2 | 4 | 3 | 12 |
| **TOTAL** | 10 | 8 | 14 | 10 | 42 |
| **TOTAL DOMAIN SCORE (%)** | **27,08333333** | | | | |
| **6. EDITORIAL INDEPENDENCE** | | | | | |
| 6a | 1 | 1 | 1 | 1 | 4 |
| 6b | 1 | 1 | 1 | 1 | 4 |
| **TOTAL** | 2 | 2 | 2 | 2 | 8 |
| **TOTAL DOMAIN SCORE (%)** | **0** | | | | |

| **AGREE II DOMAINS** | **Guideline from advising bodies** | | | | |
| --- | --- | --- | --- | --- | --- |
|  | **EDTNA-ERCA** | | | | |
| **Country** | **Europe** | | | | |
| **Appraiser** | **Appraiser 1** | **Appraiser 2** | **Appraiser 3** | **Appraiser 4** | **TOTAL** |
| **1. SCOPE AND PURPOSE** |  |  |  |  |  |
| 1a | 7 | 5 | 4 | 5 | 21 |
| 1b | 7 | 2 | 2 | 4 | 15 |
| 1c | 7 | 5 | 4 | 5 | 21 |
| **TOTAL** | 21 | 12 | 10 | 14 | 57 |
| **TOTAL DOMAIN SCORE (%)** | **62,50** | | | | |
| **2. STAKEHOLDER INVOLVEMENT** | | | | | |
| 2a | 3 | 2 | 2 | 2 | 9 |
| 2b | 4 | 2 | 1 | 2 | 9 |
| 2c | 7 | 4 | 3 | 5 | 19 |
| **TOTAL** | 14 | 8 | 6 | 9 | 37 |
| **TOTAL DOMAIN SCORE (%)** | **34,72222222** | | | | |
| **3. RIGOUR OF DEVELOPMENT** | | | | | |
| 3a | 1 | 1 | 1 | 1 | 4 |
| 3b | 1 | 1 | 1 | 1 | 4 |
| 3c | 1 | 1 | 1 | 1 | 4 |
| 3d | 2 | 1 | 1 | 1 | 5 |
| 3e | 6 | 4 | 4 | 5 | 19 |
| 3f | 3 | 4 | 3 | 3 | 13 |
| 3g | 1 | 1 | 1 | 1 | 4 |
| 3h | 1 | 1 | 1 | 1 | 4 |
| **TOTAL** | 16 | 14 | 13 | 14 | 57 |
| **TOTAL DOMAIN SCORE (%)** | **13,02083333** | | | | |
| **4. CLARITY OF PRESENTATION** | | | | | |
| 4a | 7 | 7 | 6 | 7 | 27 |
| 4b | 7 | 5 | 6 | 6 | 24 |
| 4c | 7 | 7 | 6 | 7 | 27 |
| **TOTAL** | 21 | 19 | 18 | 20 | 78 |
| **TOTAL DOMAIN SCORE (%)** | **91,66666667** | | | | |
| **5. APPLICABILITY** | | | | | |
| 5a | 2 | 1 | 4 | 2 | 9 |
| 5b | 5 | 4 | 5 | 5 | 19 |
| 5c | 1 | 1 | 4 | 2 | 8 |
| 5d | 5 | 3 | 3 | 4 | 15 |
| **TOTAL** | 13 | 9 | 16 | 13 | 51 |
| **TOTAL DOMAIN SCORE (%)** | **36,45833333** | | | | |
| **6. EDITORIAL INDEPENDENCE** | | | | | |
| 6a | 2 | 1 | 1 | 1 | 5 |
| 6b | 2 | 1 | 1 | 1 | 5 |
| **TOTAL** | 4 | 2 | 2 | 2 | 10 |
| **TOTAL DOMAIN SCORE (%)** | **4,166666667** | | | | |

| **AGREE II DOMAINS** | **Guideline from advising bodies** | | | | |
| --- | --- | --- | --- | --- | --- |
|  | **ESPEN EN** | | | | |
| **Country** | **EUROPE** | | | | |
| **Appraiser** | **Appraiser 1** | **Appraiser 2** | **Appraiser 3** | **Appraiser 4** | **TOTAL** |
| **1. SCOPE AND PURPOSE** |  |  |  |  |  |
| 1a | 4 | 5 | 6 | 5 | 20 |
| 1b | 6 | 6 | 6 | 6 | 24 |
| 1c | 7 | 5 | 6 | 6 | 24 |
| **TOTAL** | 17 | 16 | 18 | 17 | 68 |
| **TOTAL DOMAIN SCORE (%)** | **77,78** | | | | |
| **2. STAKEHOLDER INVOLVEMENT** | | | | | |
| 2a | 6 | 3 | 2 | 4 | 15 |
| 2b | 1 | 1 | 1 | 1 | 4 |
| 2c | 6 | 7 | 1 | 5 | 19 |
| **TOTAL** | 13 | 11 | 4 | 10 | 38 |
| **TOTAL DOMAIN SCORE (%)** | **36,11111111** | | | | |
| **3. RIGOUR OF DEVELOPMENT** | | | | | |
| 3a | 1 | 2 | 1 | 1 | 5 |
| 3b | 1 | 1 | 1 | 1 | 4 |
| 3c | 1 | 1 | 1 | 1 | 4 |
| 3d | 6 | 1 | 1 | 3 | 11 |
| 3e | 5 | 5 | 6 | 5 | 21 |
| 3f | 7 | 7 | 6 | 7 | 27 |
| 3g | 2 | 3 | 1 | 2 | 8 |
| 3h | 6 | 5 | 6 | 5 | 22 |
| **TOTAL** | 29 | 25 | 23 | 25 | 102 |
| **TOTAL DOMAIN SCORE (%)** | **36,45833333** | | | | |
| **4. CLARITY OF PRESENTATION** | | | | | |
| 4a | 7 | 6 | 6 | 6 | 25 |
| 4b | 7 | 7 | 6 | 7 | 27 |
| 4c | 7 | 7 | 7 | 7 | 28 |
| **TOTAL** | 21 | 20 | 19 | 20 | 80 |
| **TOTAL DOMAIN SCORE (%)** | **94,44444444** | | | | |
| **5. APPLICABILITY** | | | | | |
| 5a | 1 | 1 | 1 | 1 | 4 |
| 5b | 1 | 1 | 1 | 1 | 4 |
| 5c | 1 | 1 | 1 | 1 | 4 |
| 5d | 1 | 2 | 3 | 2 | 8 |
| **TOTAL** | 5 | 5 | 6 | 5 | 20 |
| **TOTAL DOMAIN SCORE (%)** | **4,166666667** | | | | |
| **6. EDITORIAL INDEPENDENCE** | | | | | |
| 6a | 6 | 7 | 7 | 7 | 27 |
| 6b | 6 | 7 | 7 | 7 | 27 |
| **TOTAL** | 12 | 14 | 14 | 14 | 54 |
| **TOTAL DOMAIN SCORE (%)** | **95,83333333** | | | | |

| **AGREE II DOMAINS** | **Guideline from advising bodies** | | | | |
| --- | --- | --- | --- | --- | --- |
|  | **ESPEN PN** | | | | |
| **Country** | **EUROPE** | | | | |
| **Appraiser** | **Appraiser 1** | **Appraiser 2** | **Appraiser 3** | **Appraiser 4** | **TOTAL** |
| **1. SCOPE AND PURPOSE** |  |  |  |  |  |
| 1a | 7 | 6 | 6 | 6 | 25 |
| 1b | 7 | 6 | 6 | 6 | 25 |
| 1c | 6 | 4 | 6 | 5 | 21 |
| **TOTAL** | 20 | 16 | 18 | 17 | 71 |
| **TOTAL DOMAIN SCORE (%)** | **81,94** | | | | |
| **2. STAKEHOLDER INVOLVEMENT** | | | | | |
| 2a | 4 | 3 | 2 | 3 | 12 |
| 2b | 1 | 1 | 1 | 1 | 4 |
| 2c | 1 | 1 | 2 | 1 | 5 |
| **TOTAL** | 6 | 5 | 5 | 5 | 21 |
| **TOTAL DOMAIN SCORE (%)** | **12,5** | | | | |
| **3. RIGOUR OF DEVELOPMENT** | | | | | |
| 3a | 1 | 1 | 1 | 1 | 4 |
| 3b | 1 | 1 | 2 | 1 | 5 |
| 3c | 2 | 1 | 1 | 1 | 5 |
| 3d | 2 | 1 | 2 | 2 | 7 |
| 3e | 6 | 5 | 5 | 5 | 21 |
| 3f | 2 | 5 | 6 | 4 | 17 |
| 3g | 1 | 1 | 1 | 1 | 4 |
| 3h | 1 | 1 | 1 | 1 | 4 |
| **TOTAL** | 16 | 16 | 19 | 16 | 67 |
| **TOTAL DOMAIN SCORE (%)** | **18,22916667** | | | | |
| **4. CLARITY OF PRESENTATION** | | | | | |
| 4a | 7 | 7 | 7 | 7 | 28 |
| 4b | 7 | 7 | 6 | 7 | 27 |
| 4c | 5 | 5 | 7 | 6 | 23 |
| **TOTAL** | 19 | 19 | 20 | 20 | 78 |
| **TOTAL DOMAIN SCORE (%)** | **91,66666667** | | | | |
| **5. APPLICABILITY** | | | | | |
| 5a | 1 | 1 | 1 | 1 | 4 |
| 5b | 4 | 4 | 6 | 5 | 19 |
| 5c | 1 | 1 | 1 | 1 | 4 |
| 5d | 7 | 4 | 5 | 5 | 21 |
| **TOTAL** | 5 | 10 | 13 | 12 | 48 |
| **TOTAL DOMAIN SCORE (%)** | **33,33333333** | | | | |
| **6. EDITORIAL INDEPENDENCE** | | | | | |
| 6a | 1 | 1 | 1 | 1 | 4 |
| 6b | 2 | 2 | 1 | 2 | 7 |
| **TOTAL** | 3 | 3 | 2 | 3 | 11 |
| **TOTAL DOMAIN SCORE (%)** | **6,25** | | | | |

| **AGREE II DOMAINS** | **Guideline from advising bodies** | | | | |
| --- | --- | --- | --- | --- | --- |
|  | **GARIN** | | | | |
| **Country** | **SPAIN** | | | | |
| **Appraiser** | **Appraiser 1** | **Appraiser 2** | **Appraiser 3** | **Appraiser 4** | **TOTAL** |
| **1. SCOPE AND PURPOSE** |  |  |  |  |  |
| 1a | 7 | 6 | 6 | 6 | 25 |
| 1b | 7 | 6 | 6 | 6 | 25 |
| 1c | 7 | 6 | 5 | 6 | 24 |
| **TOTAL** | 21 | 18 | 17 | 18 | 74 |
| **TOTAL DOMAIN SCORE (%)** | **86,11** | | | | |
| **2. STAKEHOLDER INVOLVEMENT** | | | | | |
| 2a | 5 | 2 | 1 | 3 | 11 |
| 2b | 1 | 1 | 1 | 1 | 4 |
| 2c | 2 | 2 | 2 | 2 | 8 |
| **TOTAL** | 8 | 5 | 4 | 6 | 23 |
| **TOTAL DOMAIN SCORE (%)** | **15,27777778** | | | | |
| **3. RIGOUR OF DEVELOPMENT** | | | | | |
| 3a | 6 | 3 | 2 | 3 | 14 |
| 3b | 6 | 4 | 6 | 5 | 21 |
| 3c | 7 | 6 | 5 | 6 | 24 |
| 3d | 2 | 2 | 3 | 2 | 9 |
| 3e | 6 | 4 | 2 | 4 | 16 |
| 3f | 7 | 7 | 6 | 7 | 27 |
| 3g | 3 | 2 | 1 | 2 | 8 |
| 3h | 1 | 1 | 1 | 1 | 4 |
| **TOTAL** | 38 | 29 | 26 | 30 | 123 |
| **TOTAL DOMAIN SCORE (%)** | **47,39583333** | | | | |
| **4. CLARITY OF PRESENTATION** | | | | | |
| 4a | 7 | 7 | 6 | 7 | 27 |
| 4b | 7 | 6 | 6 | 6 | 25 |
| 4c | 7 | 7 | 7 | 7 | 28 |
| **TOTAL** | 21 | 20 | 19 | 20 | 80 |
| **TOTAL DOMAIN SCORE (%)** | **94,44444444** | | | | |
| **5. APPLICABILITY** | | | | | |
| 5a | 1 | 1 | 1 | 1 | 4 |
| 5b | 3 | 5 | 4 | 4 | 16 |
| 5c | 1 | 1 | 2 | 1 | 5 |
| 5d | 3 | 3 | 5 | 4 | 15 |
| **TOTAL** | 8 | 10 | 12 | 10 | 40 |
| **TOTAL DOMAIN SCORE (%)** | **25** | | | | |
| **6. EDITORIAL INDEPENDENCE** | | | | | |
| 6a | 7 | 4 | 6 | 6 | 23 |
| 6b | 1 | 1 | 1 | 1 | 4 |
| **TOTAL** | 8 | 5 | 7 | 7 | 27 |
| **TOTAL DOMAIN SCORE (%)** | **39,58333333** | | | | |

| **AGREE II DOMAINS** | **Guideline from advising bodies** | | | | |
| --- | --- | --- | --- | --- | --- |
|  | **KDOQI** | | | | |
| **Country** | **International** | | | | |
| **Appraiser** | **Appraiser 1** | **Appraiser 2** | **Appraiser 3** | **Appraiser 4** | **TOTAL** |
| **1. SCOPE AND PURPOSE** |  |  |  |  |  |
| 1a | 7 | 6 | 6 | 6 | 25 |
| 1b | 7 | 7 | 7 | 7 | 28 |
| 1c | 6 | 6 | 5 | 6 | 23 |
| **TOTAL** | 20 | 19 | 18 | 19 | 76 |
| **TOTAL DOMAIN SCORE (%)** | **88,89** | | | | |
| **2. STAKEHOLDER INVOLVEMENT** | | | | | |
| 2a | 7 | 7 | 7 | 7 | 28 |
| 2b | 1 | 2 | 1 | 1 | 5 |
| 2c | 6 | 2 | 4 | 4 | 16 |
| **TOTAL** | 14 | 11 | 12 | 12 | 49 |
| **TOTAL DOMAIN SCORE (%)** | **51,38888889** | | | | |
| **3. RIGOUR OF DEVELOPMENT** | | | | | |
| 3a | 6 | 7 | 7 | 7 | 27 |
| 3b | 7 | 7 | 7 | 7 | 28 |
| 3c | 6 | 5 | 7 | 6 | 24 |
| 3d | 6 | 5 | 6 | 6 | 23 |
| 3e | 3 | 1 | 3 | 2 | 9 |
| 3f | 7 | 7 | 7 | 7 | 28 |
| 3g | 7 | 7 | 7 | 7 | 28 |
| 3h | 1 | 1 | 1 | 1 | 4 |
| **TOTAL** | 43 | 40 | 45 | 43 | 171 |
| **TOTAL DOMAIN SCORE (%)** | **72,39583333** | | | | |
| **4. CLARITY OF PRESENTATION** | | | | | |
| 4a | 7 | 7 | 7 | 7 | 28 |
| 4b | 7 | 6 | 7 | 7 | 27 |
| 4c | 7 | 7 | 7 | 7 | 28 |
| **TOTAL** | 21 | 20 | 21 | 21 | 83 |
| **TOTAL DOMAIN SCORE (%)** | **98,61111111** | | | | |
| **5. APPLICABILITY** | | | | | |
| 5a | 3 | 2 | 1 | 2 | 8 |
| 5b | 7 | 6 | 7 | 7 | 27 |
| 5c | 5 | 3 | 4 | 4 | 16 |
| 5d | 7 | 6 | 6 | 6 | 25 |
| **TOTAL** | 22 | 17 | 18 | 19 | 76 |
| **TOTAL DOMAIN SCORE (%)** | **62,5** | | | | |
| **6. EDITORIAL INDEPENDENCE** | | | | | |
| 6a | 6 | 6 | 6 | 6 | 24 |
| 6b | 7 | 7 | 7 | 7 | 28 |
| **TOTAL** | 13 | 13 | 13 | 13 | 52 |
| **TOTAL DOMAIN SCORE (%)** | **91,66666667** | | | | |

| **AGREE II DOMAINS** | **Guideline from advising bodies** | | | | |
| --- | --- | --- | --- | --- | --- |
|  | **SIN-ANDID-ANED** | | | | |
| **Country** | **ITALY** | | | | |
| **Appraiser** | **Appraiser 1** | **Appraiser 2** | **Appraiser 3** | **Appraiser 4** | **TOTAL** |
| **1. SCOPE AND PURPOSE** |  |  |  |  |  |
| 1a | 5 | 4 | 4 | 4 | 17 |
| 1b | 6 | 4 | 4 | 5 | 19 |
| 1c | 4 | 3 | 5 | 4 | 16 |
| **TOTAL** | 15 | 11 | 13 | 13 | 52 |
| **TOTAL DOMAIN SCORE (%)** | **55,56** | | | | |
| **2. STAKEHOLDER INVOLVEMENT** | | | | | |
| 2a | 5 | 3 | 7 | 5 | 20 |
| 2b | 2 | 3 | 6 | 4 | 15 |
| 2c | 1 | 1 | 4 | 2 | 8 |
| **TOTAL** | 8 | 7 | 17 | 11 | 43 |
| **TOTAL DOMAIN SCORE (%)** | **43,05555556** | | | | |
| **3. RIGOUR OF DEVELOPMENT** | | | | | |
| 3a | 1 | 1 | 1 | 1 | 4 |
| 3b | 1 | 1 | 1 | 1 | 4 |
| 3c | 1 | 1 | 1 | 1 | 4 |
| 3d | 1 | 1 | 1 | 1 | 4 |
| 3e | 1 | 3 | 1 | 2 | 7 |
| 3f | 1 | 4 | 1 | 2 | 8 |
| 3g | 1 | 1 | 1 | 1 | 4 |
| 3h | 1 | 1 | 1 | 1 | 4 |
| **TOTAL** | 8 | 13 | 8 | 1 | 39 |
| **TOTAL DOMAIN SCORE (%)** | **3,645833333** | | | | |
| **4. CLARITY OF PRESENTATION** | | | | | |
| 4a | 5 | 4 | 1 | 3 | 13 |
| 4b | 2 | 1 | 2 | 2 | 7 |
| 4c | 4 | 3 | 5 | 4 | 16 |
| **TOTAL** | 11 | 8 | 8 | 9 | 36 |
| **TOTAL DOMAIN SCORE (%)** | **33,33333333** | | | | |
| **5. APPLICABILITY** | | | | | |
| 5a | 1 | 1 | 1 | **1** | 4 |
| 5b | 1 | 1 | 1 | **1** | 4 |
| 5c | 1 | 1 | 1 | **1** | 4 |
| 5d | 1 | 1 | 1 | **1** | 4 |
| **TOTAL** | 4 | 4 | 4 | 4 | 16 |
| **TOTAL DOMAIN SCORE (%)** | **0** | | | | |
| **6. EDITORIAL INDEPENDENCE** | | | | | |
| 6a | 4 | 4 | 1 | 3 | 12 |
| 6b | 7 | 7 | 1 | 5 | 20 |
| **TOTAL** | 11 | 11 | 2 | 8 | 32 |
| **TOTAL DOMAIN SCORE (%)** | **50** | | | | |

| **AGREE II DOMAINS** | **Guideline from advising bodies** | | | | |
| --- | --- | --- | --- | --- | --- |
|  | **The Renal Association** | | | | |
| **Country** | **UK** | | | | |
| **Appraiser** | **Appraiser 1** | **Appraiser 2** | **Appraiser 3** | **Appraiser 4** | **TOTAL** |
| **1. SCOPE AND PURPOSE** |  |  |  |  |  |
| 1a | 2 | 1 | 2 | 2 | 7 |
| 1b | 1 | 1 | 1 | 1 | 4 |
| 1c | 2 | 1 | 1 | 1 | 5 |
| **TOTAL** | 5 | 3 | 4 | 4 | 16 |
| **TOTAL DOMAIN SCORE (%)** | **5,56** | | | | |
| **2. STAKEHOLDER INVOLVEMENT** | | | | | |
| 2a | 3 | 2 | 2 | 2 | 9 |
| 2b | 1 | 1 | 1 | 1 | 4 |
| 2c | 3 | 5 | 1 | 3 | 12 |
| **TOTAL** | 7 | 8 | 4 | 6 | 25 |
| **TOTAL DOMAIN SCORE (%)** | **18,05555556** | | | | |
| **3. RIGOUR OF DEVELOPMENT** | | | | | |
| 3a | 5 | 4 | 4 | 4 | 17 |
| 3b | 1 | 1 | 1 | 1 | 4 |
| 3c | 1 | 1 | 7 | 3 | 12 |
| 3d | 5 | 1 | 6 | 3 | 15 |
| 3e | 1 | 1 | 3 | 2 | 7 |
| 3f | 4 | 4 | 7 | 5 | 20 |
| 3g | 3 | 3 | 7 | 4 | 17 |
| 3h | 1 | 1 | 1 | 1 | 4 |
| **TOTAL** | 21 | 16 | 36 | 23 | 96 |
| **TOTAL DOMAIN SCORE (%)** | **33,33333333** | | | | |
| **4. CLARITY OF PRESENTATION** | | | | | |
| 4a | 7 | 4 | 7 | 6 | 24 |
| 4b | 7 | 3 | 3 | 4 | 17 |
| 4c | 6 | 6 | 3 | 5 | 20 |
| **TOTAL** | 20 | 13 | 13 | 15 | 61 |
| **TOTAL DOMAIN SCORE (%)** | **68,05555556** | | | | |
| **5. APPLICABILITY** | | | | | |
| 5a | 1 | 1 | 1 | 1 | 4 |
| 5b | 1 | 1 | 1 | 1 | 4 |
| 5c | 1 | 1 | 1 | 1 | 4 |
| 5d | 1 | 1 | 1 | 1 | 4 |
| **TOTAL** | 4 | 4 | 4 | 4 | 16 |
| **TOTAL DOMAIN SCORE (%)** | **0** | | | | |
| **6. EDITORIAL INDEPENDENCE** | | | | | |
| 6a | 1 | 1 | 1 | 1 | 4 |
| 6b | 7 | 7 | 6 | 7 | 27 |
| **TOTAL** | 8 | 8 | 7 | 8 | 31 |
| **TOTAL DOMAIN SCORE (%)** | **47,91666667** | | | | |
