# Supplementary figures and images for: AGREEing on Nutritional Management of Patients with CKD—A Quality Appraisal of the Available Guidelines
Source: Nutrients. 2021 Feb 15;13(2):624. doi: 10.3390/nu13020624 (PMC7918946; doi:10.3390/nu13020624)

**Supplementary Figure 1**: Summary of AGREE II tool result of each domain


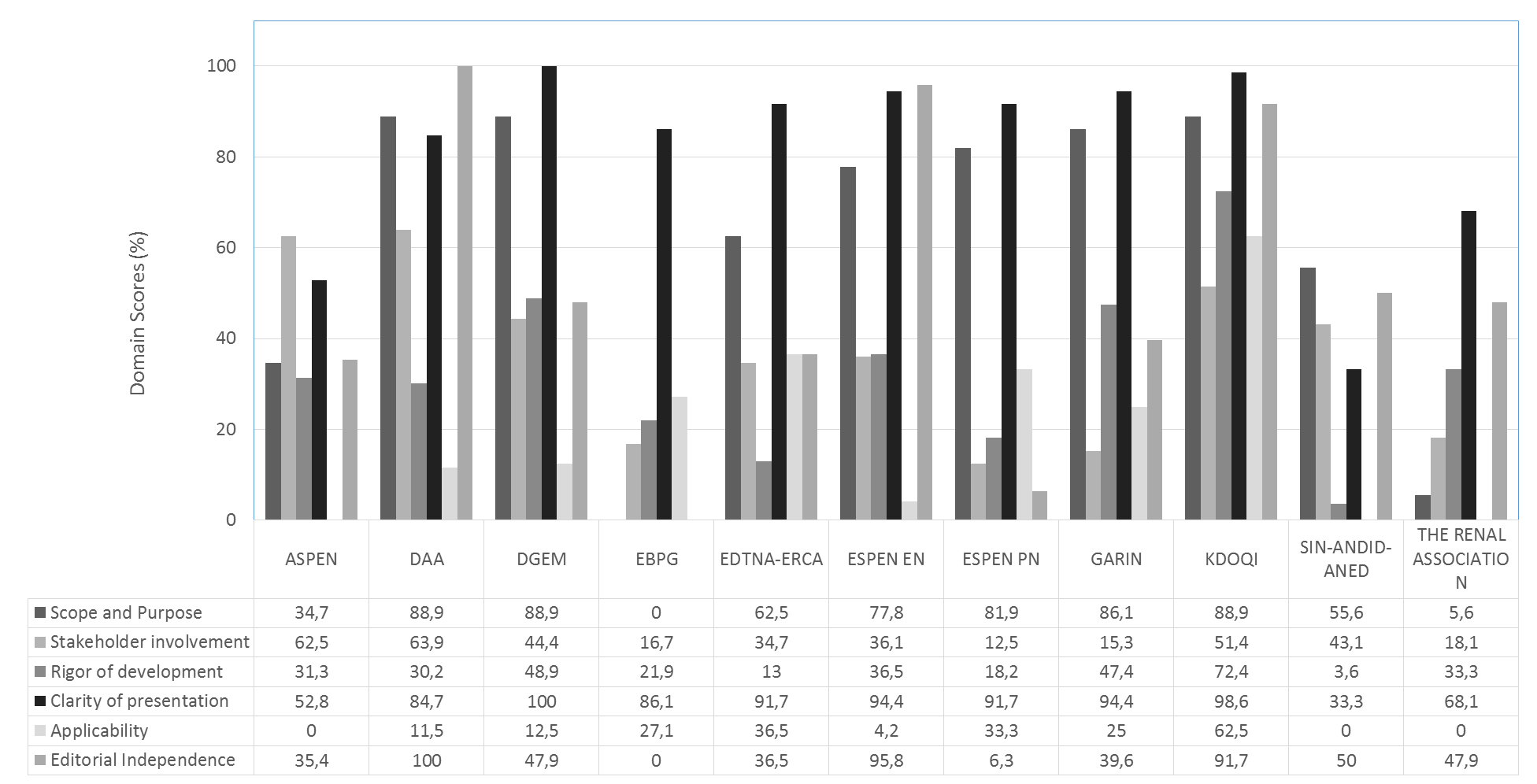

Supplement: Supplementary file 1 [file nutrients-13-00624-s001.zip › nutrients-1050150-supplementary materials/Supplementary Figure S1.docx]
